# Volatile chemical composition of *Octoblepharum albidum* Hedw. (Bryophyta) from the Brazilian Amazon

**DOI:** 10.1186/s13065-022-00872-4

**Published:** 2022-10-09

**Authors:** Raynon Joel Monteiro Alves, Thyago Gonçalves Miranda, Rafaela Oliveira Pinheiro, Wandson Braamcamp de Souza Pinheiro, Eloisa Helena de Aguiar Andrade, Ana Cláudia Caldeira Tavares-Martins

**Affiliations:** 1 Universidade Federal Do Pará, Belém, PA 66075-110 Brasil; 2 Museu Paraense Emílio Goeldi, Belém, PA 66077-830 Brasil; 3 Universidade Do Estado Do Pará, Belém, PA 66050-540 Brasil

**Keywords:** Bryophytes, Biotechnology, Calymperaceae, Bioactive compounds, Phytochemistry

## Abstract

Bryophytes have a variety of bioactive compounds that can be used in biotechnological processes. The objective of this study was to know the volatile chemical composition of *Octoblepharum albidum* Hedw. from the Amazon and investigate its association with possible bioactive effects on insects. The volatile concentrate of *O. albidum* was obtained by micro-scale simultaneous distillation–extraction and analyzed by gas chromatography coupled to mass spectrometry and the identification of the compounds was based on system libraries and specialized literature. Twelve organic compounds (92.44% of the total) were identified. Hexadecanoic acid, oleic acid, E-isoeugenol, 1-octen-3-ol, and stearic acid were the major compounds. Most of the compounds have already been reported from bryophytes, while others have an unprecedented occurrence in the group. All identified compounds have biological activities reported in the literature and may participate in plant defense mechanisms against insects, causing mortality or developmental inhibition. In this study, we describe for the first time the volatile chemical composition of *O. albidum* from Brazil and provide evidence that this species is a source of bioactive compounds. The identified compounds have been reported in the literature to cause mortality or affect the biological parameters of insects, what suggests the possibility of their usage in the formulation of bioinsecticides.

## Introduction

Bryophytes are avascular plant species taxonomically placed between algae and pteridophytes, divided into three groups: mosses (Bryophyta), liverworts (Marchantiophyta) and hornworts (Anthocerotophyta) [1]. These plants have a high diversity of chemical compounds with biological activities often reported in the literature. These natural bioactive compounds are considered “chemical weapons” that function as a defense mechanism against fungi, bacteria and insects and compensate for the absence of the usual mechanical protection (thick cuticle and bark layers) present in other plant groups [2–4].

It is estimated that there are about 12,700 species of mosses worldwide. They are conspicuous floristic components of all terrestrial habitats, from Antarctica to the exuberant tropical forests [5, 6]. The moss family Calymperaceae is composed of pantropical and strictly endemic taxa that occur in disparate environments, such as for example, from the Pacific islands to the Amazon basin [7]. There are 28 genera distributed worldwide and four genera in Brazil, namely, *Calymperes* Sw. ex Weber, *Leucophanes* Brid., *Syrrhopodon* Schwägr., and *Octoblepharum* Hedw. [8].

In Brazil, the few phytochemical studies carried out with *Calymperes lonchophyllum* Schwägr. and *Octoblepharum albidum* Hedw. have provided satisfactory results with potential application in biotechnological processes, such as the production of antibiotics and pesticides [9, 10].

*Octoblepharum albidum* occurs in all phytogeographic domains of Brazil (the Amazon, Caatinga, Cerrado, Pantanal, Atlantic Forest and Pampa) [8, 11]. This species is characterized by medium-sized plants that grow in loose tufts or cushions, rarely solitary, presenting an opaque whitish coloration, often vinaceous at the base of the leaves; short, simple, radiculose stems; spreading leaves with ligulate shape, broad oval-obovate, sometimes concave base, with apiculate apex, with entire margins below and serrated margins at the apex, presenting 2–3 layers of leucocysts above and below a medial band of chlorocysts in cross-sectional view; and sporophytes with a short seta and an ovoid-cylindric capsule [8].

Ten classes of bioactive secondary compounds were found in the ethanolic extract of *O. albidum*, which showed antibacterial activity alone and in association with antibiotics, mainly against *Escherichia coli* and *Klebsiella pneumoniae* [10]. The ethanolic extract of this species had insecticidal activity at different concentrations, killing most third-instar *Spodoptera frugiperda* (JE Smith, 1797) (Lepidoptera: Noctuidae) caterpillars in cowpea (*Vigna unguiculata* (L.) Walp.) leaves, especially from 48 h of the experiment onwards (ALVES et al., unpublished data).

Bryophyte species contain primary and secondary compounds that can be easily extracted and used as effective insecticides in agriculture [12]. Therefore, considering the lack of research on the volatile chemical composition of *O. albidum* and its relationship with various bioactivities, especially on compounds with potential application in bioinsecticide formulations, this study aimed to determine the volatile chemical composition of Amazonian specimens of this species and its association with possible bioactive effects on insects.

## Experimental

### Plant material and extraction procedure

Specimens of *O. albidum* were collected from trunks of live trees in domestic backyards in the state of Pará using the techniques proposed by [13]. The identification/confirmation of the species followed the taxonomic classification of [13]. As different bryophyte species often grow intermingled in clusters together with other organisms, such as invertebrates, the specimens of interest were screened and separated with the aid of a magnifying glass and tweezers. Subsequently, a fresh sample (11 g) including gametophytes and sporophytes of *O. albidum* was subjected to micro-scale simultaneous distillation–extraction (DES) in a Nickerson & Likens extraction apparatus from Chrompack, using n-pentane (3 mL) as solvent, coupled to a refrigeration system to maintain the temperature of the condenser between 5 and 10 °C for 2 h, in duplicate.

The volatile concentrate was analyzed by means of gas chromatography coupled to mass spectrometry (GC/MS) using a Shimadzu QP-2010 Plus system equipped with a Rtx-5MS capillary column (Restek Corporation, Bellefonte, PA USA) (30 m ×0.25 mm; 0.25 μm film thickness) under the following operating conditions: carrier gas: helium, with a linear velocity of 36.5 cm/s; injection type: splitless (2 μL); injector and detector temperature: 250 °C; oven temperature program: 40–60 °C (2 °C/min), 60–250 °C (3 °C/min); MS: electron impact, 70 eV; temperature of ion source and connecting parts: 220 °C. Compound identification was done by comparison of mass spectra and retention indices (RI) with those of substances in the libraries of the system (NIST) and literature data [14, 15]. The RI were obtained using the homologous series of n-alkanes (C8–C40) (Sigma-Aldrich, Milwaukee, WI, USA). The components were quantified by means of GC in a Shimadzu QP-2010 Plus instrument equipped with a flame ionization detector (FID) under the same operating conditions except for the carrier gas, which was hydrogen.

### Ethical statement

This work has authorization registered with the National System for Management of Genetic Heritage and Associated Traditional Knowledge (SISGEN), with Registration Number: A952E48.

## Results and discussion

**Fig. 1:**
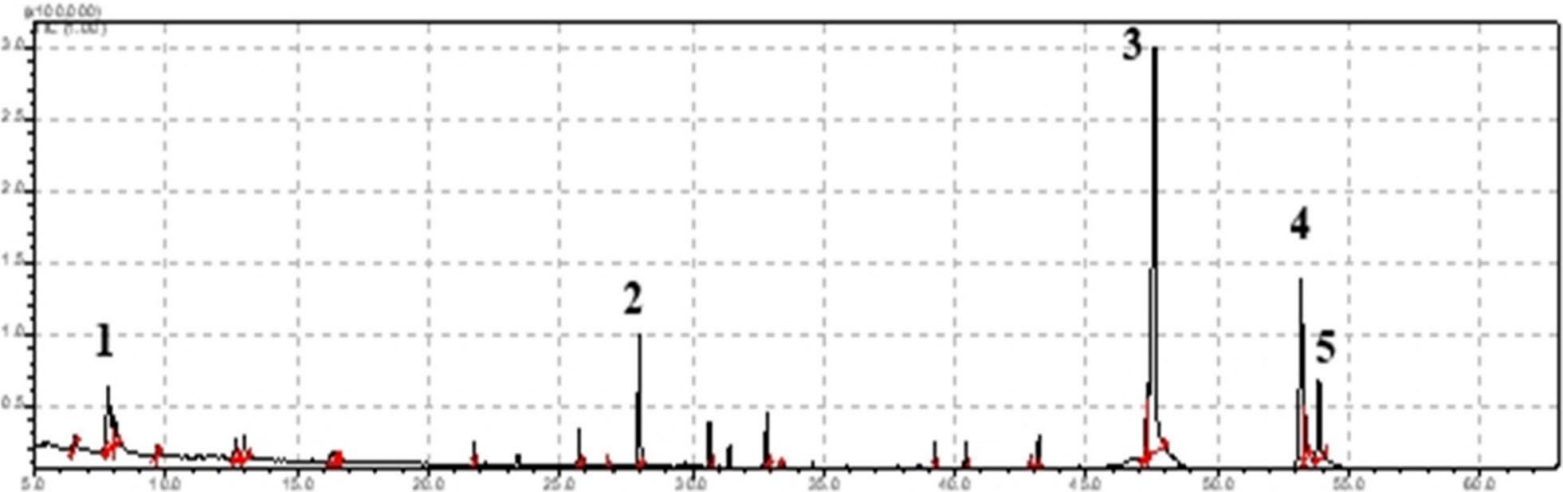
Ion chromatogram of the volatile concentrate of *Octoblepharum* albidum Hedw

Twelve organic compounds, corresponding to 92.44% of the total compounds, were identified in the volatile concentrate of *O. albidum*. The major compounds were hexadecanoic acid (43.97%), oleic acid (17.8%), E-isoeugenol (7.09%), 1-cten-3-ol (5.87%), and stearic acid (4.84%) (Table 1 and Fig. 1). The phytochemical screening of the ethanolic extract of *O. albidum* conducted by [10] identified the presence of tannins phlobaphenes, tannins pyrogallates, anthocyanins, flavones, flavonols, flavonones, aurones, proanthocyanidins, alkaloids, and terpenes. Extraction by sample enrichment probe and identification by GC-MS showed aliphatic alcohols and aldehydes (1-hexanol, 7-octen-4-ol, hexanal and nonanal) in *O. albidum*, in addition to a large amount of fatty acids, especially hexadecanoic acid [16]. Fatty acids produced by bryophytes can be saturated, monounsaturated, polyunsaturated and acetylenic and, together with triglycerides, glycolipids, phospholipids, sterols, wax esters, fatty alcohols and terpenoids, they are classified as lipids because of their hydrophobic nature [17].

**Table 1:** Chemical composition of the volatile concentrate of *Octoblepharum albidum* Hedw

Constituents	Class	RI_C_	RI_L_	%
2E-Hepten-1-ol	Alcohol	926	958	0.63
*1-octen-3-ol*	*Alcohol*	*964*	*974*	*5.87*
3-Octanone	Ketone	971	979	2.76
ni	–	1016	–	0.4
ni	–	1097	–	1.11
1-octen-3-yl acetate	Ester	1105	1110	1.33
2-methoxy-4-methyl-phenol	Phenol	1188	1188	1.91
ni	–	1300	–	0.61
ni	–	1391	–	1.79
ni	–	1416	–	0.38
*E-isoeugenol*	*Phenylpropanoid*	*1444*	*1448*	*7.09*
Methyl-p-tert-butyl-phenylacetate (T)	Carboxylic ester	1507	1497	1.9
Dodecanoic acid (lauric)	Fatty acid	1561	1565	3.23
ni		1573		0.2
ni		1724		0.76
ni		1757		1.09
ni		1826		0.34
Neophytadiene	Terpene	1835	1838	1.11
*Hexadecanoic acid (palmitic acid)*	*Fatty acid*	*1963*	*1959*	*43.97*
*(Z)-octadec-9-enoic acid (oleic acid)*	*Fatty acid*	*2141*	*2141*	*17.8*
*Octadecanoic acid (stearic acid)*	*Fatty acid*	*2163*	*2165*	*4.84*

*RI*_*C*_ calculated retention index *RI*_*L*-_ literature retention index main constituents in italics *Ni* not identified

Fatty acids (hexadecanoic, oleic, stearic, and dodecanoic acid) stood out in the volatile concentrate of *O. albidum* (Table 1 and Fig. 1). Hexadecanoic and stearic acids are some common saturated fatty acids synthesized by bryophytes, and several poly- and monounsaturated fatty acids such as oleic acid are abundant in all bryophyte species [17]. Some of these compounds isolated from bryophytes, such as hexadecanoic and dodecanoic acids, have insecticidal activity against *Sitophilus granarius* (Coleoptera: Curculionidae) [12]. The viability of *S. frugiperda* larvae was reduced under the influence of oleic, stearic and hexadecanoic acids, tested separately, and the latter was the most effective [18]. Linoleic acid was more toxic than oleic acid and stearic acid against *Spodoptera littoralis* (Boisduval) (Lepidoptera: Noctuidae) larvae, and when mixed with commercial insecticidal products, linoleic acid and oleic acid had, respectively, potentiation and additive effects, interfering with the biological parameters of the insects [19].

Hexadecanoic acid can be converted into unsaturated fatty acid, possibly contributing, at least partially, to the synthesis of longer fatty acids, such as eicosapentaenoic acid, which can deter herbivory [20]. Eicosapentaenoic acid (C20) and arachidonic acid (C20) are produced in high concentrations by bryophytes, but are rarely found in other plant groups [21]. These two very-long-chain acids are precursors for the biosynthesis of some C8 aromatic fatty alcohols, such as 1-octen-3-ol, octan-3-one and octan-3-ol, as observed after mechanical stress in *Marchantia polymorpha* L. [22]. *Neckeropsis undulata* (Hedw.) Reichardt has also been reported to produce large amounts of octen-3-ol after injury, which suggests that rapid formation of this compound in wounded sites of mosses and liverworts is necessary for defense against pathogens and herbivores [23, 24]. 1-octen-3-ol and 3-Octanone corresponded to 5.87% and 2.76%, respectively, of the volatile concentrate of *O. albidum* (Table 1 and Fig. 1).

E-isoeugenol was one of the main compounds found in *O. albidum* in this study, with a concentration of 7.09%, representing the first record in mosses (Table 1 and Fig. 1). Eugenol and its derivatives have a pungent and spicy aroma and have already been reported in liverwort species and in the essential oils of angiosperms [25–27]. Isoeugenol showed contact toxicity against *Sitophilus zeamais* Motschulsky and *Tribolium castaneum* (Herbst) (both Coleoptera: Curculionidae) and reduced the growth rate and food consumption of larvae and/or adults of the two species in a concentration-dependent manner [28]. Eugenol and isoeugenol caused high larval mortality, dissuaded oviposition of females, and reduced egg hatchability of *Tuta absoluta* Meyrick (Lepidoptera: Gelechiidae) [29]. Isoeugenol caused mortality of *Drosophila melanogaster* Meigen (Diptera; Drosophilidae) and higher volumes of this compound decreased the flying capacity of flies [30].

2-methoxy-4-methyl-phenol and methyl-p-tert-butyl phenylacetate were also identified in *O. albidum*, with concentrations of 1.91% and 1.90%, respectively. The occurrence of these compounds is unprecedented in bryophytes (Table 1 and Fig. 1). 2-methoxy-4-methyl-phenol contained in the liquid smoke of coconut fiber is one of the phenolic compounds used in the insecticide industry, whose tested product caused 60–80% mortality of *Epilachna sparsa* (Hbst.) (Coleoptera: Coccinellidae) [31]. Methyl-p-tert-butyl phenylacetate was the only compound identified in the ethanolic extract of seeds of *Annona squamosa* Linn. and was observed to cause a mortality rate of more than 50% of brown planthopper *Nilaparvata lugens* (Stål) (Homoptera: Delphacidae) [32].

1-octen-3-yl acetate was found in the volatile concentrate of *O. albidum* at a concentration of 1.33% (Table 1 and Fig. 1). In the literature, this ester has been found only in liverworts, such as *Conocephalum, Marchantia, Dumortiera, Pellia, Plagiochila*, and *Wiesnerella* species, being the compound responsible for the mushroom odor of these bryophytes [22, 33–35]. 1-octen-3-yl acetate was the predominant C8 volatile present in intact thalli of *M. polymorpha*, while tissue disruption resulted in the conversion of the acetate to 1-octen-3-ol [22]. C8 volatiles are, in general, known to perform a signaling function when in the vapor phase [22]. 1-octen-3-yl *β*-primeveroside extracted from soybean (*Glycine max* L.) has been considered to function as a form of storage of volatile 1-octen-3-ol for immediate response against mechanical damage to leaf tissues, suggesting its participation in plant defense mechanism [36].

The diterpene neophytadiene was present at a concentration of 1.11% in the volatile composition of *O. albidum*. The presence of this compound has already been documented in other moss and liverwort species [37–39] (Table 1 and Fig. 1). Neophytadiene is usually found in all green plants, as a product of chlorophyll degradation [40]. This compound was one of the most active obtained from the bio-oil produced from the residues of greenhouse tomato plants, exhibiting toxicity against *Leptinotarsa decemlineata* Say*.* (Coleoptera: Chrysomelidae) larvae [41]. In turn, 2E-Hepten-1-ol was the minor component in the volatile concentrate of *O. albidum* (0.63%) (Table 1 and Fig. 1). Similar aliphatic alcohols, such as 2-hepten-1-ol and 4-hepten-1-ol, have already been reported from moss species, and other alcohols, such as 1-hexanol and 7- octen-4-ol, have been recorded in *O. albidum*, showing that this class of compounds is part of the oxylipin volatilome of these bryophytes [16]. Oxylipines are oxygenated fatty acids that participate in the defense of bryophytes, being produced in abundance after tissue injury or pathogen attack [42].

## Conclusion

The volatile composition of *O. albidum* from Brazil is reported for the first time here, using GC/MS. We showed evidence that this specie is a source of fatty acids, phenolic compounds, alcohols, ketones, esters, and terpenes. Further, the occurrence of some compounds in *O. albidum* is unprecedented in mosses and even in bryophytes, as for example E-isoeugenol, 2-methoxy-4-methyl-phenol, and methyl-p-tert-butyl-phenylacetate. The identified compounds have been reported in the literature to cause mortality or affect the biological parameters of insects, what suggests the possibility of their usage in the formulation of bioinsecticides.

## Data Availability

The datasets used and/or analysed during the current study are available from the corresponding author on reasonable request.
